# Using gene expression signatures to identify novel treatment strategies in gulf war illness

**DOI:** 10.1186/s12920-015-0111-3

**Published:** 2015-07-09

**Authors:** Travis J.A. Craddock, Jeanna M. Harvey, Lubov Nathanson, Zachary M. Barnes, Nancy G. Klimas, Mary Ann Fletcher, Gordon Broderick

**Affiliations:** Center for Psychological Studies, Nova Southeastern University, Fort Lauderdale, USA; Graduate School of Computer and Information Sciences, Nova Southeastern University, Fort Lauderdale, USA; Institute for Neuro-Immune Medicine, Nova Southeastern University, 3440 South University Drive, Fort Lauderdale, FL 33328 USA; College of Osteopathic Medicine, Nova Southeastern University, Fort Lauderdale, USA; Department of Medicine, University of Alberta, Edmonton, Canada; Miller School of Medicine, University of Miami, Miami, USA; Miami Veterans Affairs Medical Center, Miami, USA; Diabetes Research Institute, University of Miami, Miami, USA

**Keywords:** Gulf war illness, Systems biology, Bioinformatics, Drug repurposing, Pharmacogenomics, Complex chronic illness

## Abstract

**Background:**

Gulf War Illness (GWI) is a complex multi-symptom disorder that affects up to one in three veterans of this 1991 conflict and for which no effective treatment has been found. Discovering novel treatment strategies for such a complex chronic illness is extremely expensive, carries a high probability of failure and a lengthy cycle time. Repurposing Food and Drug Administration approved drugs offers a cost-effective solution with a significantly abbreviated timeline.

**Methods:**

Here, we explore drug re-purposing opportunities in GWI by combining systems biology and bioinformatics techniques with pharmacogenomic information to find overlapping elements in gene expression linking GWI to successfully treated diseases. Gene modules were defined based on cellular function and their activation estimated from the differential expression of each module’s constituent genes. These gene modules were then cross-referenced with drug atlas and pharmacogenomic databases to identify agents currently used successfully for treatment in other diseases. To explore the clinical use of these drugs in illnesses similar to GWI we compared gene expression patterns in modules that were significantly expressed in GWI with expression patterns in those same modules in other illnesses.

**Results:**

We found 19 functional modules with significantly altered gene expression patterns in GWI. Within these modules, 45 genes were documented drug targets. Illnesses with highly correlated gene expression patterns overlapping considerably with GWI were found in 18 of the disease conditions studied. Brain, muscular and autoimmune disorders composed the bulk of these.

**Conclusion:**

Of the associated drugs, immunosuppressants currently used in treating rheumatoid arthritis, and hormone based therapies were identified as the best available candidates for treating GWI symptoms.

**Electronic supplementary material:**

The online version of this article (doi:10.1186/s12920-015-0111-3) contains supplementary material, which is available to authorized users.

## Background

Novel drug discovery is a costly, high-risk and extremely time-consuming enterprise. Costing in the range of millions to billions of dollars [1–4], the process can take up to 15 years to complete with one of every two novel drug candidates failing in the later stages [5]. As such, the repurposing of Food and Drug Administration (FDA) approved drugs offers a fast cost effective solution for discovering novel treatments. This is all the more true for conditions with no known treatments.

Gulf War Illness (GWI) is but one example of a complex chronic illness with no known cure and which requires long-term treatment and monitoring. This both prolongs patient suffering and increases the financial burden of the illness, for the individual, the family and society. GWI is a multi-symptom disorder exhibiting a complex constellation of symptoms that include fatigue, musculoskeletal pain, and cognitive dysfunction [6]. Since returning from the first Gulf War over 20 years ago investments of nearly $1 billion have been made to Gulf War Veterans health [6], however there is still no effective treatment available for the nearly 250,000 veterans with GWI. Though the cause and illness mechanisms of GWI are largely unknown, a leading hypothesis points to the involvement of neuroinflammatory cascade possibly triggered by exposure to battlefield toxins and exacerbated by stress [7–9]. Thus, this illness has far-reaching consequences and the development of effective treatments promises to benefit not only this patient population but a host of others as well.

In an attempt to identify candidate treatment avenues for GWI involving currently approved pharmacological agents we used a combination of systems biology and bioinformatics techniques combined with pharmacogenomic information to compare gene expression patterns in GWI to known drug targets and expression patterns found in a set of human diseases. We identified a specific set of functional gene modules with altered expression in GWI pointing to the cellular processes affected in this disorder. Significant correlations between gene expression profiles in these gene modules were found between GWI and other disease conditions. Cross-referencing of disease and drug information in pharmacogenomic databases with druggable genes in these functional modules ultimately revealed novel drug repurposing avenues for the treatment of GWI.

## Methods

### GWI cohort

As part of a larger ongoing study a subset of GWI male subjects (*n* = 17) and healthy but sedentary Gulf War era veterans (*n* = 22) were recruited from the Miami Veterans Administration Medical Centers, clinics and the local veteran community between April 2006 and May 2008. All subjects were comparable in age, body mass index (BMI), and ethnicity. Subjects were male and ranged in age between 30 and 55. Inclusion criteria was derived from Fukuda *et al.* [10], and consisted in identifying veterans deployed to the theater of operations between August 8, 1990 and July 31, 1991, with one or more symptoms present after 6 months from at least 2 of the following: fatigue; mood and cognitive complaints; and musculoskeletal complaints. Subjects were in good health prior to 1990, and had no current exclusionary diagnoses [11]. Medications that could have impacted immune function were excluded. Use of the Fukuda definition in GWI is supported by Collins *et al.* [12]. Additional details may be found in Broderick *et al.* [13].

All subjects signed an informed consent approved by the Institutional Review Board of the University of Miami. Ethics review and approval for data analysis was also obtained by the IRB of the University of Alberta.

### Gene expression

Blood was drawn at rest at comparable times of day from each subject during the April 2006 to May 2008 period. At each blood draw three 8-mL tubes of blood were collected in CPT vacutainers (B-D- Biosciences, San Jose, CA). The peripheral blood mononuclear cells (PBMC) were isolated and stored in liquid nitrogen under conditions designed to maintain viability. Specifically, whole blood was added to Ficoll-Paque, centrifuged at 1000 g for 25 min. PBMC’s were isolated from the PBMC ring atop the Ficoll layer into a separate tube, centrifuged at 300 g for 10 min, then re-suspended in PBS. Cells were then counted using a Beckman Coulter viCell, and cryopreserved in freezing media (temperature lowered 1 ^o^C per minute until -80 ^o^C).

Total RNA was extracted using TRI Reagent (Molecular Research Center, Cincinnati, OH) following the manufacturer’s protocol. The quality and quantity of RNA was assessed using the Agilent Bioanalyzer 2100 RNA 6000 Nano Kit (Agilent Technologies, CA). From each sample, 300 ng of total RNA was converted into cDNA by reverse transcription using a T7-oligo(dT) primer and the Affymetrix 3′ IVT Express Kit (Affymetrix, Santa Clara, CA) according to standard manufacturer protocol. The generated cDNA was purified using the GeneChip Sample Cleaning Module (Affymetrix) and labeled cRNA was generated by *in vitro* transcription using the biotinylated nucleotide mix. This was then purified with the Cleaning Module and quantified using the Nanodrop ND-1000 spectrophotometer (NanoDrop Technologies, Inc., Wilmington, DE USA). In each preparation 11 μg cRNA was fragmented in Fragmentation Buffer (Affymetrix) in a final reaction volume of 25 μl.

Hybridization, washing, staining and scanning were done using Affymetrix GeneChip instruments (Hybridization Oven 640, Fluidics Station 450Dx, Scanner GCS3000Dx) and Affymetrix Human U133 2.0 arrays (Affymetrix) as per manufacturer’s standards. Microarray image files (.cel data) were generated using the Affymetrix GCOS software tool with default microarray analysis parameters to provide overall within chip normalization of the image intensity distribution. The quality parameters that were monitored besides cRNA total yield and cRNA A260/A280 ratio included: (i) background noise (Q value), (ii) percentage of present called probe sets, (iii) scaling factor, (iv) information about exogenous Bacillus subtilis control transcripts from the Affymetrix Poly-A control kit (lys, phe, thr, and dap), and (v) the ratio of intensities of 3′ probes to 5′ probes for a housekeeping gene (GAPDH).

To generate a broad comparison group the Gene Expression Omnibus (GEO) DataSets [14, 15] and data from Suthram *et al.* [16] were used to obtain a set of gene expression profiles describing a number of human disease conditions. We restricted our selection to include only those sets in which both disease and a corresponding healthy control group were measured in the same cell type or tissue in the same experimental conditions. Sets that included different exposure times, exposure concentrations or multiple cell/tissue types were each treated as a separate disease condition. For consistency, and to avoid complications arising due to cross platform comparisons, datasets were restricted to the Affymetrix Gene Chip Human Genome U133A, U133 Plus 2.0, U133A 2.0 and U95 Version 2 arrays, to align with our GWI gene expression data. All diseases affecting male subjects in the GEO database meeting these criteria were included in this study. Overall, this resulted in 101 human disease gene expression datasets (Additional file 1: Table S1). The disease profiles selected provide a comprehensive set of diseases and include various cancers, neurodegenerative diseases, autoimmune illnesses, chemical exposures, viral infections, neurological and neuromuscular disorders.

### Data transformation and normalization

Gene expression data was Log2 transformed then normalized using a Z-score transformation for each microarray sample to allow for the direct comparison of values across various samples and diseases.

### Gene functional modules

Here we use 4,620 functional modules defined by Suthram *et al.* [16] from the human protein-protein interaction network. Individual genes i in module j were compared between GWI subjects and healthy controls using an unpaired *t*-test to generate a T-Score, G_ij_. Differential module activity (MA_j_) was determined using the average of the absolute T-Score values for all N_j_ genes in a given module, j, such that:

1 $$ M{A}_j=\frac{{\displaystyle {\sum}_{i=1}^{N_j}\left|{G}_i\right|}}{N_j} $$

This is a modification of the module activity described in [16] were the signed T-score was used. When averaging over signed T-scores equal but opposite scores will tend to cancel, resulting in no module activity, when in fact there are genes significantly over or under expressed. Here the absolute value was used in the differential module activity to capture both up and down-expressed genes. A threshold of MA = 1.5, corresponding to a p-value of ~0.1, was chosen as a liberal cutoff value to identify differentially expressed gene modules. Only modules with MA values above threshold were taken to be differentially expressed in GWI and considered for further analysis, all others were discarded.

### Individual gene expression analysis

The 202 genes in the 19 modules with MA values above threshold were individually compared between GWI and controls. Individual genes i were compared between GWI subjects and healthy controls using an unpaired *t*-test. To account for multiple comparisons false discovery rates (FDR) were then calculated for each comparison from these resulting p-values using the procedure introduced by Storey [17]. Genes with FDR of less than or equal to 0.05 were taken to be significantly different in GWI compared to control. Fold change was calculated by calculating the ratio of average gene expression between GWI and health controls for non-Log2 transformed data. Positive values were taken to indicate the fold-increase while ratios that were less than 1 were inverted and given a negative value to denote the fold-decrease. An absolute fold change of 1.5 was taken as a cutoff [18].

### Mining the pharmacogenomic knowledge base (PharmGKB) database

Genes from modules affected in GWI were screened against the PharmGKB database (8.1.2015) [19] to find gene-drug and gene-disease relationships supported with pharmacogenomics research reported in the literature.

### Pathway-based functional annotation of gene modules

Functional annotation of gene modules with gene-drug relations was performed using the ConsensusPathDB [20–22] to provide biological pathway information for each gene set. Over-representation analysis [20] incorporating the Kyoto Encyclopedia of Genes and Genomes (KEGG) (73.0) [23], Netpath (1.1.2015) [24], the Small Molecule Pathway Database (SMPDB) (8.1.2015) [25], the Integrating Network Objects with Hierarchies (INOH) (1.1.2015) [26], Biocarta (2009_05_12) [27], Humancyc (18.5) [28], Signalink (8.1.2015) [29], Edinburgh human metabolic network (Ehmn) (1.1.2015) [30], Reactome (51) [31], PharmGKB (8.1.2015) [19], Wikipathways (9.1.2015) [32] and the Pathway Interaction Database (PID) (2014_02_14) [33] pathway sets was used to interpret the function of druggable gene modules. Here the significance of the observed overlap between the gene module and the members of known pathways, compared to random expectations, was calculated based on the hypergeometric distribution. A minimum overlap of 2 genes between the gene module and the pathway set at a p-value cutoff 0.01 was required. Specifically, the p-value is calculated as the probability of randomly finding k or more successes from the population in N total draws. Thus, small p-values indicate a greater overlap than expected by chance. Pathway sets containing the majority of the druggable genes, the highest number of module genes overall, and the lowest p-value were taken as the functional annotation of the module. Pathway annotation was performed only to provide biological pathway information for each gene module set. All subsequent analysis was performed on these gene module sets constructed on the bases of human protein-protein interactions [16] and not on the bases of known pathway membership.

### Gene module alignment across illnesses

To provide a quantitative measure of similarity in module expression between GWI and another disease a partial Spearman correlation was calculated between the T-Score values of genes compared between select diseases (Additional file 1: Table S1) and their controls within each of the modules preferentially expressed in GWI [16]. Partial correlation measures the degree of association between two random variables, while removing the effect of a set of controlling random variables. Partial Spearman correlation was used to assess disease similarity while explicitly factoring out dependencies between different gene expression experiments [16]. The Partial Spearman correlation coefficient between GWI (*G*) and a disease condition (*D*), *r*_*GD,C*_, was conditioned on the T-Score values between the control samples (*C*), corresponding to each comparator disease, such that:

2 $$ {r}_{GD,C}=\frac{r_{GD}-{r}_{GC}{r}_{DC}}{\sqrt{\left(1-{r}_{GC}^2\right)}\sqrt{\left(1-{r}_{DC}^2\right)}} $$

with the generic Spearman correlation coefficient, *r*_*GD*_, given as:

3 $$ {r}_{GD}=\frac{{\displaystyle {\sum}_{i=1}^{N_j}\left({G}_i-\overline{G}\right)\left({D}_i-\overline{D}\right)}}{\sqrt{{\displaystyle {\sum}_{i=1}^{N_j}{\left({G}_i-\overline{G}\right)}^2}}\sqrt{{\displaystyle {\sum}_{i=1}^{N_j}{\left({D}_i-\overline{D}\right)}^2}}} $$

where *G*_*i*_ the ranked TScores between GWI and controls, *D*_*i*_ the ranked TScores between a given human disease condition and controls, and *C*_*i*_ the ranked TScores between between GWI and human disease condition control groups for the *i*^*th*^ gene in module *j* containing *N*_*j*_ genes. Note that the terms in the numerator of Equation (2) are given by Equation (3) with substitutions for *G*, *D*, and *C* appropriately as labeled. Here in Equation (3) the barred terms ($\overline{G},\overline{D},\overline{C}$) denote the averaging of all genes in a given module. Correlations with *p*-values ≤ 0.01 were considered statistically significant. Additionally, as we were looking for similarity between modules, inverse correlations were not considered. Partial correlations were calculated between GWI and the 101 disease conditions (Additional file 1: Table S1) in the GWI affected modules.

### Significance of correlation

A random background distribution of disease correlations across each module was created to determine if module correlations between GWI and other diseases were significantly different from random chance. Gene to module assignments were first randomized, while preserving the total number of modules, the number of genes per module, and the number of modules to which each gene belongs as in Suthram *et al.* [16]. Then disease and control datasets for the disease conditions were randomly shuffled before calculating partial correlations with intact GWI datasets. This process was performed 100 times for each module to generate the random background distributions. The cumulative distribution of the random background correlations per module was used to calculate the p-value of the true correlations in each module. FDR was calculated for each correlation from these resulting p-values using the procedure introduced by Storey [17]. Correlation values with FDR of less than or equal to 0.05 were taken to be significantly different from random background. All others were removed.

## Results

### Affected genes and modules

**Fig. 1:**
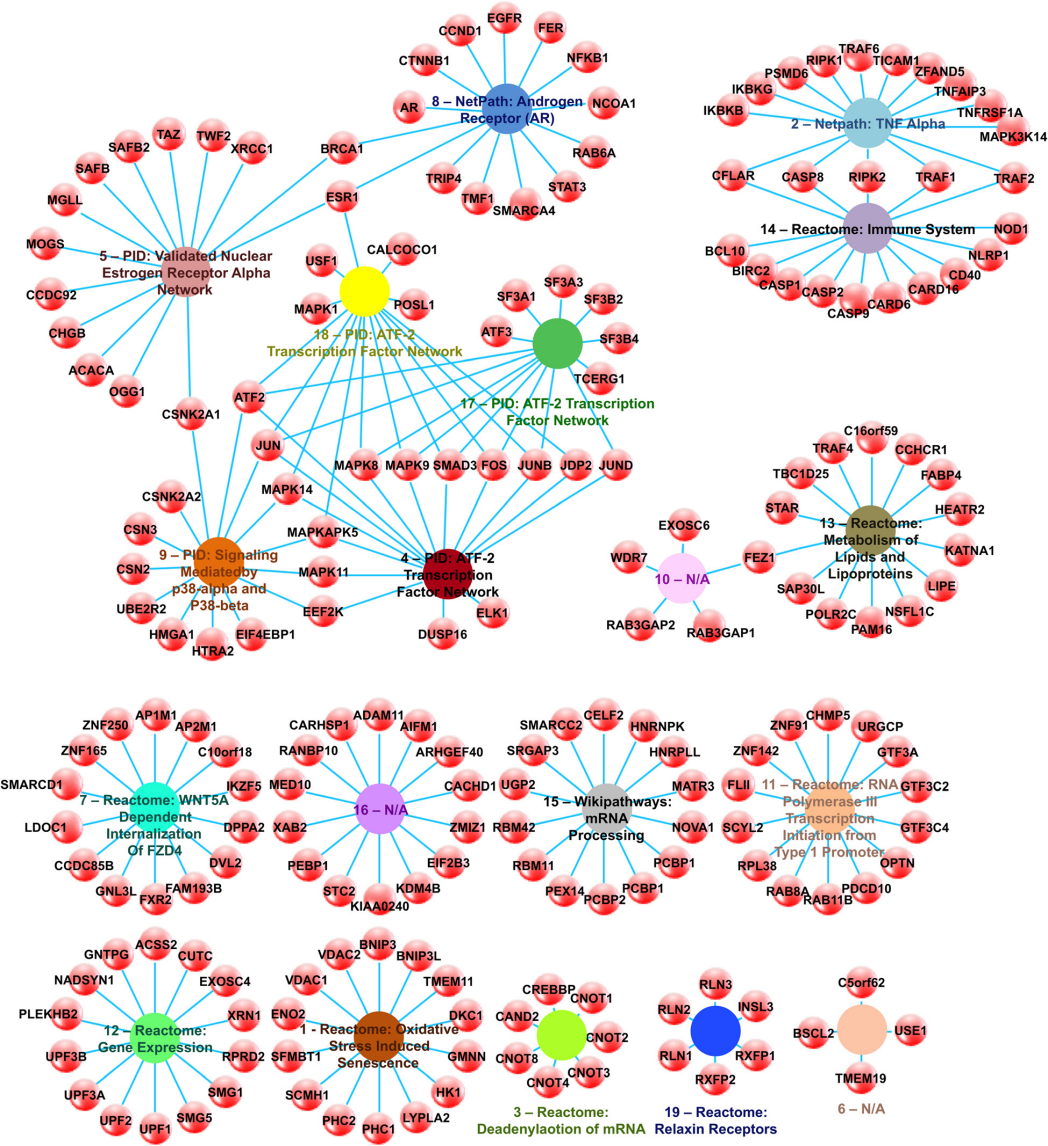
GWI Affected Modules. Network illustrating modules (colored spheres) identified as differentially expressed in GWI and their corresponding gene (red spheres) associations. Edges denote gene membership within a given module consistent with previous methodologies [84]

A work flow illustrating the above methods is provided in Additional file 2: Figure S1. In this work we found 19 of the 4,620 gene expression modules (~0.4 %) to be differentially expressed in GWI compared to healthy controls. Many of the modules (10 of 19) identified as being affected in GWI shared common characteristics and were clustered together across overlapping member genes (Fig. 1). Pathway-based functional annotation of these gene modules provided biological pathway information for each gene set (Fig. 1) at a significance of *p* < 0.1 and at a false discovery rate of q < 0.1. Statistics for the pathway-based functional annotation can be found in Additional file 3: Table S2. These 19 modules were comprised of 202 individual genes. Of these 202 genes 54 were found to have a significant change of 1.5 fold or more (See Additional file 4: Table S3).

### Drug targetable affected genes

**Fig. 2:**
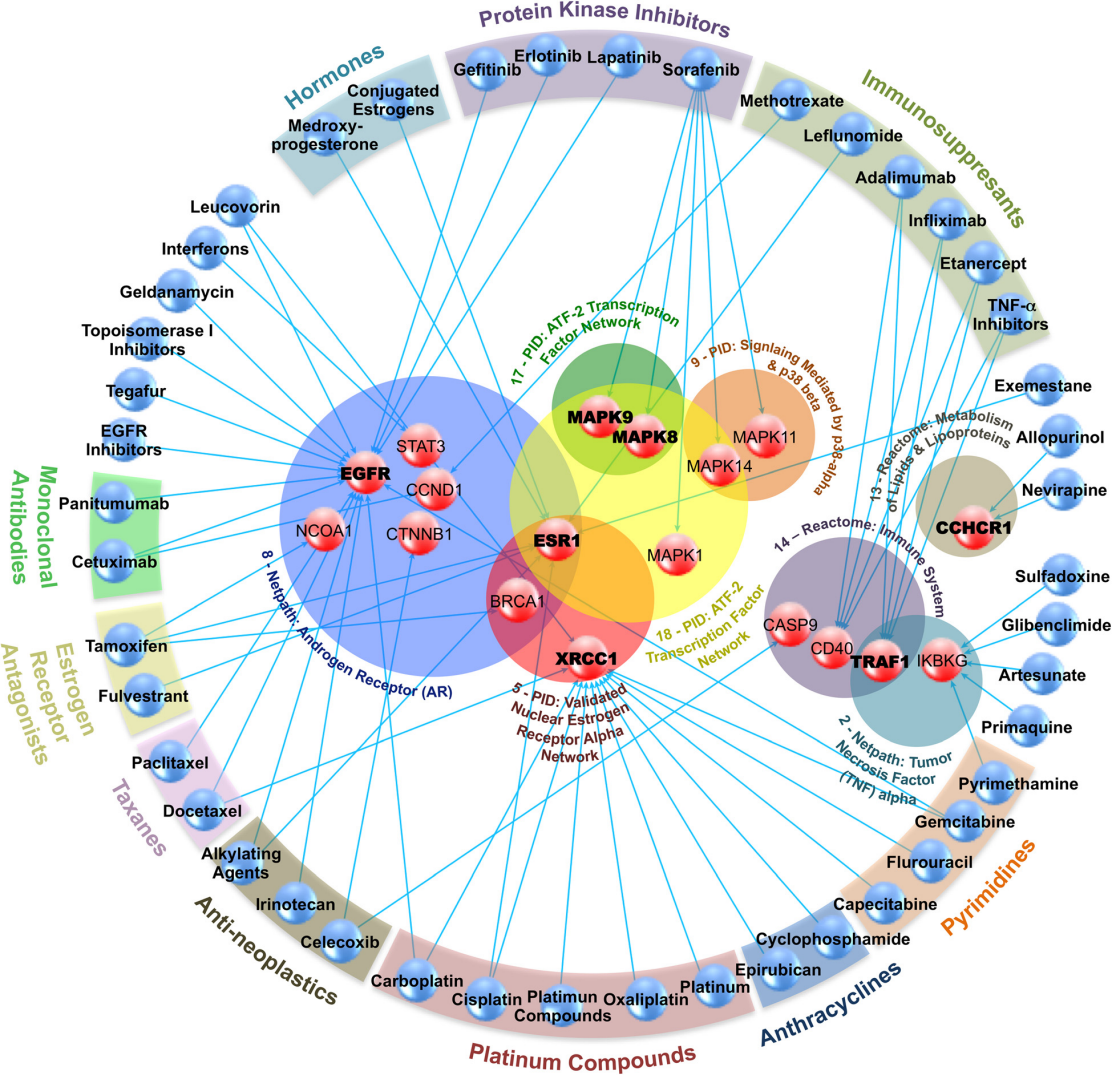
GWI Affected Drug Targetable Genes. Network illustrating PharmGKB database (8.1.2015) [19] documented gene-drug (red-blue spheres) associations for genes in GWI affected modules (overlapping colored circles) arranged according to drug families (colored bars). Gene module number scheme refers to Fig. 1. Genes presented in boldface show a significant (*p* ≤ 0.05) difference in GWI compared to controls with an absolute fold change greater than 1.5

Cross-referencing the 202 genes in the 19 GWI affected modules to drugs known to affect these genes using the PharmGKB database (8.1.2015) [19] yielded 10 distinct categories of treatment, plus several uncategorized drugs (Fig. 2). Of the compounds affecting the 45 targetable genes 6 were immunosuppressants (13 %), 5 were platinum compounds (11 %), 4 were protein kinase inhibitors (9 %), 4 were pyrimidines (9 %), 3 were anti-neoplastics (7 %), 2 were hormones (4 %), 2 were monoclonal antibodies (4 %), 2 were anti-estrogens (4 %), 2 were taxanes (4 %), and 2 were anthracyclines (4 %), with the remainder being single entries from unidentified drug classes. All identified drug treatable genes were found in 8 of the 19 identified GWI affected modules. Of the 45 targetable genes, only 7 showed a significant fold change in GWI: CCHCR1, EGFR, ESR1, MAPK8, MAPK9, TRAF1 and XRCC1 (Fig. 2 and Additional file 4: Table S3).

### Pathway representation in druggable gene modules

Of these 8 drug targetable GWI modules, 86.7 % of the genes in module 2 belonged to the Tumor Necrosis Factor (TNF) alpha signaling pathway as annotated in the Netpath database (1.1.2015) [24] (Additional file 3: Table S2). This pathway includes both drug targetable genes, TNF receptor-associated factor 1 (TRAF1) and the inhibitor of kappa light polypeptide gene enhancer in B-cells, kinase gamma (IKGBG). Overlapping with this module is module 14, which embodies much of the Immune System pathway as annotated in the Reactome database (51) [31]. Overall, 73.3 % of the genes in module 14 are associated with this pathway including the caspase 9, apoptosis-related cysteine peptidase (CASP9) gene, and the CD40 molecule, TNF receptor superfamily member 5 (CD40) gene.

Modules 5, 8, 9, 17, and 18 overlap to form a cluster of targetable genes. Module 5 was annotated as the PID (2014_02_14) [33] pathway for the Validated Nuclear Estrogen Receptor Alpha Network. While only 21.4 % of the genes in module 5 are included in this pathway, two of the three drug targets identified are among these, namely the breast cancer 1, early onset (BRCA1), and estrogen receptor 1 (ESR1) genes. The third druggable target, the X-ray repair cross-complementing protein 1 (XRCC1) gene, however was not included in the Validated Nuclear Estrogen Receptor Alpha Network. The Netpath (1.1.2015) [24] Androgen Receptor (AR) pathway was representative of the genes present in module 8. A total of 62.4 % of the genes in this module were found in the AR pathway, including 6 of the 7 drug targetable genes: BRCA1, cyclin D1 (CCND1), epidermal growth factor receptor (EGFR), nuclear receptor coactivator 1 (NCOA1) catenin (cadherin-associated protein), beta 1, 88 kDa (CTNNB1), and signal transducer and activator of transcription 3 (acute-phase response factor) (STAT3). Only ESR1 was not affiliated with the AR pathway. The PID (2014_02_14) [33] pathway for Signaling Mediated by p38-alpha & p38 beta was assigned as the annotation for module 9. Both drug targetable genes, mitogen activated protein kinases 11 and 14, are included among the 42.9 % of module 9 genes found in this pathway. Genes in both modules 17 and 18 were identified as part of the PID (2014_02_14) [33] ATF-2 Transcription Factor Network. This pathway contains 57.1 % and 66.7 % of the genes in module 17 and 18, respectively, including all of the identified drug targets for these modules, namely MAPK 1, 8, 9 and 14, and ESR1.

Finally, module 13 was enriched in genes from the Reactome database (51) [31] Metabolism of Lipids & Lipoproteins pathway. Only 21.4 % of the genes in the module are included in this pathway, and the coiled-coil alpha-helical rod protein 1 (CCHCR1) drug targetable gene is not among them making this isolated pathway uninformative for the selection of treatment repurposing in GWI.

### Similarity of GWI to known human diseases

**Fig. 3:**
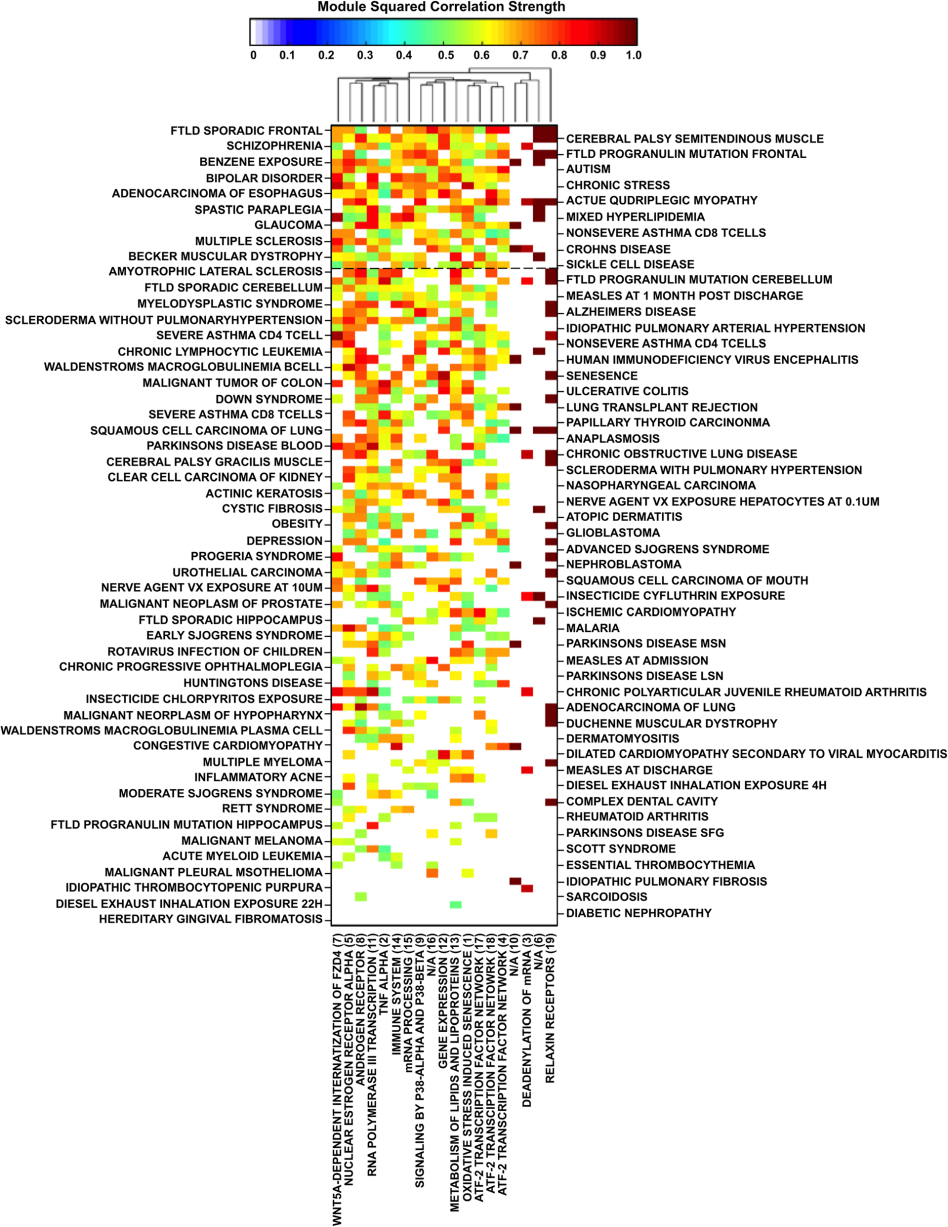
Matrix of squared correlation values for GWI with all human diseases (Additional file 1: Table S1) in all affected modules (Fig. 1) with values between 0 and 1 according to the color bar. Diseases are arranged in decreasing GA value and modules refer to those presented in Fig. 1 and Additional file 3: Table S2. Dashed black line represent the GA 50 % mark. Modules are clustered hierarchically using a Euclidean distance metric and average linkage to generate the hierarchical tree

We compared the 19 gene expression patterns in modules that were significantly expressed in GWI with expression patterns in those same modules in other illnesses to find similarities between GWI and these illnesses (Fig. 3). The average of all significant correlation values across all GWI affected modules for a given disease gives a Global Alignment (GA) value describing the similarity between GWI and the disease in question (Additional file 1: Table S1). A GA value of 100 % indicates perfect correlation across all 19 affected modules. For example, Actinic Keratosis was found to be significantly correlated with GWI in 6 of the 19 modules. The sum of the 6 correlations values is 7.24 hence its GA value is 7.24/19 = 38.1 %. All the correlations averaged in the calculation of the GA are significant (*p* < 0.01; FDR < 0.05) giving a measure of the degree to which each illness aligns with GWI. Eighteen disease conditions were found to have GA values of 50 % or greater. These overwhelmingly represented features shared by GWI with a number of neurological and neurodegenerative illnesses such as various forms of brain inflammation and degeneration including multiple sclerosis as well as muscle degeneration, paraplegia and myopathy. Interestingly these also included pathologic response to benzene exposure (GA = 68 %) and chronic activation of the stress response axis (GA = 65 %).

### Refinement of drug targets through illness similarity

**Fig. 4:**
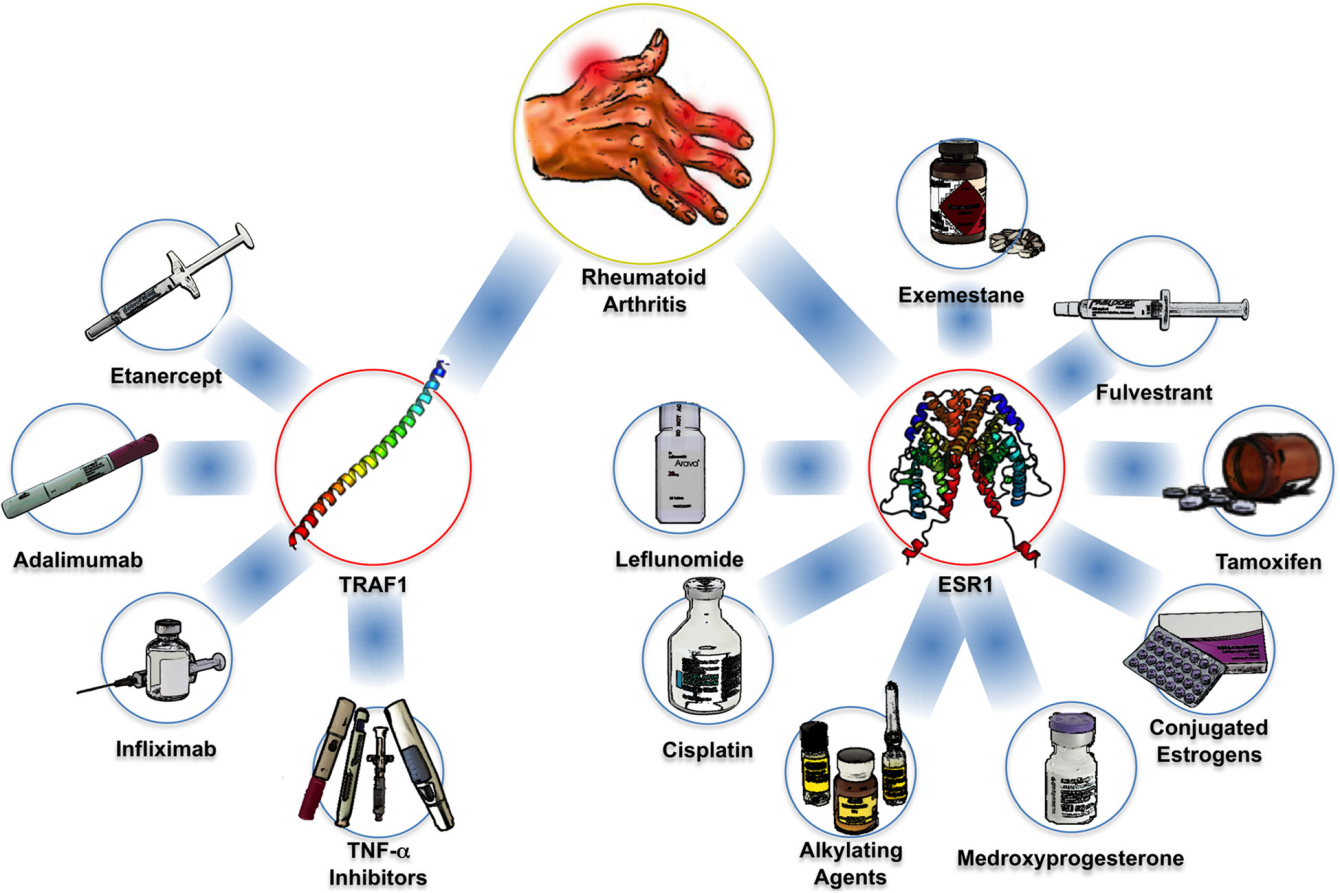
Summary of Results. Disease – Gene – Drug associations identified via the PharmGKB database (8.1.2015) [19]. Squared correlation values of 0.542, 0.634, 0.551 for Rheumatoid Arthritis (RA) with GWI in the Netpath (1.1.2015) [24]: Tumor Necrosis Factor (TNF) alpha, PID (2014_02_14) [33]: Validated Nuclear Estrogen Receptor Alpha Network, and PID (2014_02_14) [33]: ATF-2 Transcription Factor Network pathways, respectively

To focus on established clinical use of potential treatments we further refined our initial list of 8 druggable GWI gene modules through the cross-referencing of disease and drug information in the PharmGKB database (8.1.2015) [19]. Illnesses showing gene expression profiles significantly correlated with GWI in the 8 druggable gene modules were cross-referenced with drug agents specifically used for their successful treatment. We found one illness overlapping with specific druggable components of GWI, namely rheumatoid arthritis (RA) (GA = 16 %) (Fig. 3). The correlations between GWI and RA occur along the Netpath (1.1.2015) [24] Tumor Necrosis Factor (TNF) alpha pathway (R^2^ = 0.55), the PID (2014_02_14) [33] annotated Validated Nuclear Estrogen Receptor Alpha Network (R^2^ = 0.63), and ATF-2 Transcription Factor Network (R^2^ = 0.54) pathways. The gene encoding TRAF1 in the Netpath (1.1.2015) [24] Tumor Necrosis Factor (TNF) alpha pathway is targeted with the specific monoclonal antibodies against TNF-α infliximab and adalimumab, the chimeric protein etanercept, and an overall class of TNF-α inhibitors in general (Fig. 4). The drug-targetable ESR1 gene identified in RA is annotated in PID (2014_02_14) [33] as part of both the Validated Nuclear Estrogen Receptor Alpha Network, and the ATF-2 Transcription Factor Network pathways. Drugs identified in relation to these pathways and RA are the immunosuppressant leflunomide, the platinum compound cisplatin, general anti-neoplastic alkylating agents, and the estrogen modulating agents medroxprogesterone, conjugated estrogens, tamoxifen, fluvestrant, and exemestane (Fig. 4).

## Discussion

GWI is a complex multi-symptom illness, which manifests with cognitive dysfunction, fatigue, and musculoskeletal pain [6]. At this time it is unclear whether GWI represents several syndromes, or one syndrome with several subtypes. While stratification of the case defined GWI subjects into subtypes based on shared clusters of symptoms is an active area of research which may provide improved resolution on underlying gene functional involvement, this issue will only be resolved when objective markers are firmly established. The analysis presented here is a step towards this goal.

Cognitive difficulties, such as memory problems, constitute one of the most common unexplained impairments reported in GWI [34]. Recent work examining brain changes in GWI support an underlying neurobiological underpinning to these problems. Results show that participants with GWI perform significantly slower and less accurately on working memory tasks than matched healthy veterans, and that this decrease correlates with lower levels of activity in prefrontal brain regions [35]. Fatigue and pain, other commonly reported symptoms associated with GWI have been linked to alterations in the brain’s white matter in GWI subjects [36, 37]. Memory problems, as well as mood disorder, have also been linked to mild inflammation and degenerations in the hippocampus in a mouse model of GWI [38]. Our findings, indicate that over a third (38.9 %) of the diseases aligning with greater than 50 % of the GWI affected modules are localized to the central nervous system.

Musculoskeletal pain is another frequently reported symptom of GWI [34]. A higher proportion of veterans of the Persian Gulf War of 1991 reported symptoms of muscle and joint pain than a military comparison group [39]. This chronic pain has been linked to abnormal central processing of sensory and painful stimuli in GWI subjects in brain areas normally serving sensory perception, and threat and arousal, as well as in thalamocortical circuits and cerebellum [40]. GWI subjects suffering from chronic musculoskeletal pain also experience greater naturally occurring muscle pain during exercise compared to healthy veterans and become more sensitive to pain stimuli following acute exercise suggesting that acute exercise augments the central nervous system sensitivity to sensory information [41]. In this work we find that more than 1 in 5 (22 %) of the diseases, which overlap in gene expression with GWI, are muscular disorders.

Evidence is mounting that there is also a significant immune component to GWI. Ongoing Th1-type immune activation, as measured by intracellular production of cytokines in peripheral blood, appears to be symptomatic in afflicted Gulf War Veterans when compared to healthy counterparts [42]. More recently, this finding has been confirmed while also suggesting that this may occur in the more complex context of a mixed Th1:Th2 response [43]. In addition to this, our group previously reported altered gene expression associated with NK cell function and decreased NK cell cytotoxicity in GWI subjects [44]. Abnormal regulation of the immune system can result in autoimmune diseases [45], and allergies [46]. Like GWI, these illnesses are characterized by abnormal resting levels of immune cells, cytokines and circulating hormones. Our results indicate that close to 1 in 6 (17 %) of the overlapping diseases are autoimmune disorders.

While gene expression in GWI was measured in PBMC, gene expression in some of the other human illnesses was obtained from brain tissue (e.g. hippocampus in FTLD, substantia nigra in Parkinson’s). Although these measures of pathway activation were expressed in different tissue, substantial alignment between gene expression in blood and brain biopsies has been shown in Parkinson’s, Alzheimer’s and Huntington’s [48–50] supporting the relevance of this profiling. When considered collectively, these findings support an illness model whereby GWI might be described primarily as a central nervous system disorder manifesting with musculoskeletal problems that is potentially fueled by a dysregulation of immunity. Central to this would be an ongoing neuroinflammatory process.

A common issue in pathway analysis is that genes shared between overlapping functional modules and pathways may cause an inflated or biased p-value of statistical significance for some annotations. In such situations highly influential genes that are shared across multiple pathways may bias the identification of some pathways unfairly. The current analysis is not immune to this problem. As such it is pertinent to examine the results in the context of the specific illness in question, and the individual genes highlighted by the analysis. Pathway annotation of the drug treatable GWI affected modules revealed two major pathway clusters. The first is dominated by the Netpath (1.1.2015) [24] annotated pathway for Tumor Necrosis Factor (TNF) alpha and the Reactome (51) [31] Immune System pathway. Overall, this cluster of pathways suggest that immune signaling, particularly TNF-α signaling, are prime targets for repurposed drug treatment in GWI. Projecting this onto known illnesses with similar pathway involvement pointed to commonalities with RA. The relation between GWI and RA in the Netpath (1.1.2015) [24]: Tumor Necrosis Factor (TNF) alpha pathway identifies the gene encoding TRAF1 in conjunction with several TNF-α inhibitors. Suppressed activity of TNF receptor and apoptotic pathways [13], and higher responsiveness of TNF-α [43, 45] have previously been identified in GWI subjects by our group, consistent with the current findings, suggesting an autoimmune component to this illness. TNF-α blockers, such as infliximab, adalimumab and etanercept, are currently approved by the FDA for the treatment of chronic inflammatory diseases such as RA, Crohn’s disease and ankylosing spondylitis. While, such treatments may affect immune function, inducing autoantibodies leading to conditions such as drug-induced lupus, there is low risk of infliximab, etanercept, and adalimumab in association with drug-induced lupus despite the higher incidence of autoantibodies with its use [47]. As chronic inflammation is a suggested component of GWI pathophysiology [51], these compounds may provide a promising novel treatment avenue for GWI, however further tests are required.

The second pathway cluster central to GWI that was identified here is comprised of the Netpath (1.1.2015) [24] Androgen Receptor (AR) pathway and PID (2014_02_14) [33] Validated Nuclear Estrogen Receptor Alpha Network as well as PID (2014_02_14) [33] pathways for the ATF-2 Transcription Factor Network, and Signaling Mediated by p38-alpha and p38-beta. This large cluster is targetable by the majority of identified drug classes including immunosuppressants, protein kinase inhibitors, hormone treatments, estrogen receptor antagonists, monoclonal antibodies, taxanes, anti-neoplastics and platinum compounds. The p-38 MAPKs participate in signaling cascades that control cellular responses to immune signals and stress, while ATF-2 is normally activated in response to signals that converge on the stress-activated p38 MAPKs. Both the PID (2014_02_14) [33] pathway Validated Nuclear Estrogen Receptor Alpha Network pathway and Netpath (1.1.2015) [24]: Androgen Receptor (AR) pathway highlight sex steroid hormone pathways. As androgens are the precursor of all estrogens, and both estrogen [52] and androgen [53] receptor activity is mediated by p38 signaling, this pathway cluster suggests that sex steroid signaling, as mediated by p38, may also be prime targets for repurposed drug treatment in GWI.

Estrogen is also a potent, albeit complex, modulator of inflammation [54]. The estrogen receptor 1 (ESR1) gene is identified in relation to RA in both the PID database (2014_02_14) [33] Validated Nuclear Estrogen Receptor Alpha Network, and ATF-2 Transcription Factor Network pathways. Leflunomide, an immunosuppressive disease-modifying anti-rheumatic drug whose efficacy is modulated by ESR1 gene polymorphisms [55], is the only drug identified with a direct use in active moderate to severe rheumatoid arthritis. The remainder of the drug candidates identified in association with ESR1 and RA are most commonly used in the treatment and prevention of estrogen receptor positive cancers. Cisplatin, a platinum-containing anti-cancer drug that binds DNA resulting in apoptosis, also has immunosuppressive effects and has been shown to be effective in managing RA in a recent single case study [56]. Alkylating agents, drugs that modulate the immune system such as cyclophosphamide, are sometimes used to treat severe cases of rheumatoid arthritis [57]. The remaining candidate drugs associated with RA and ESR1 directly modulate estrogen signaling. Tamoxifen and fulvestrant, are selective estrogen receptor modulators (SERMs), that act as antagonists in breast and agonists in other tissue. Tamoxifen has been shown to exert anti-inflammatory effects in human RA cell cultures [58, 59]. Additionally, other SERMs currently show promise in treating RA in mouse models of both genders [60, 61]. However, estrogen receptor antagonists have also been reported to induce RA like symptoms in female subjects [62] (female mice), [63]. Likewise exemestane, an irreversible steroidal aromatase inactivator, has been suggested to induce or reveal RA in female subjects [64–66]. The discrepancy in effects may be attributable to the influence of gender on tamoxifen-induced biochemical changes [67, 68].

Though based on gene expression in peripheral blood, these results are nonetheless consistent with the involvement of a neuroinflammatory component in GWI [6]. Estrogen plays a significant role in inflammation in general [54], and neuroinflammation specifically [69], mediating via astrocytes neuroprotective effects on clinical function, inflammation in the central nervous system, and axonal loss [70]. Furthermore, estrogen receptor α moderates the cytokine- and chemokine-mediated neuroinflammatory response [71]. SERMs, including tamoxifen and fulvestrant, appear to have an important anti-inflammatory role [72] and are thus a promising treatment avenue for GWI. However, as we have found GWI subjects under maximal exercise challenge exhibit increased activation of pathways involving NF-κB [13] the tendency of SERMs to increase activity of the nuclear NFκB complex [73] must be taken into consideration. Evidence also suggests a role for estrogen and estrogen receptors in Amyotrophic Lateral Sclerosis (ALS). The incidence and prevalence of ALS are greater in men than in women [74] and evidence supports the notion that endogenous female hormones have a neuroprotective effect on motor neurons [75, 76]. This is of import as Gulf War veterans have been shown to develop amyotrophic lateral sclerosis (ALS) at twice the rate of non-deployed era veterans [77, 78]. Our analysis is consistent with this showing a GA value of 49 % between ALS and GWI. Early phase II trials of tamoxifen in ALS have demonstrated preliminary efficacy [79, 80], and therefore may be of therapeutic benefit for ALS like symptoms associated with GWI. Additionally, estrogen receptor agonists and estrogen have been shown to attenuate TNF-α induced apoptosis in motor neurons [75, 81]. While SERMs and TNF-α inhibitors provide novel treatment avenues for GWI, further analysis must be performed.

## Conclusions

Collectively this work supports a treatment strategy that would target both immune and sex hormone signaling in GWI. Interestingly this aligns with our recent studies of altered neuroendocrine-immune homeostasis in GWI using computational models of known immune, stress (hypothalamic-pituitary-adrenal) and sex (hypothalamic-pituitary-gonadal) hormone signaling [82, 83]. In both these studies we found the cross-talk between immune and sex steroid signaling could in principle support alternate neuroendocrine-immune regulatory modes that resemble chronic GWI-associated signatures. Specifically we found evidence in this analysis that GWI may be associated with dysregulation in genetic pathways involved in immune signaling, particularly TNF-α, signaling, and sex steroid signaling, as mediated by p38 MAPKs. When compared to a set of human disease conditions GWI most closely resembles brain disorders with musculoskeletal problems and dysregulation of immunity, consistent with previously observed reports. Overall, while specific drugs have been highlighted the general findings of our study suggest the use of TNF-α immunosuppressive agents and SERMs, either individually or in conjunction, in the treatment of GWI. Further analysis of these treatment avenues will ultimately reveal their efficacy.

## Additional files

Additional file 1: Table S1. Tables listing disease conditions and GEO series numbers. Additional file 2: Figure S1. A figure providing a graphical summary of the methods. Additional file 3: Table S2. Tables listing pathway-based functional annotation statistics. Additional file 4: Table S3. Tables listing individual gene comparison statistics.
