# Supplementary material for: Using gene expression signatures to identify novel treatment strategies in gulf war illness
Source: BMC Med Genomics. 2015 Jul 9;8:36. doi: 10.1186/s12920-015-0111-3 (PMC4495687; doi:10.1186/s12920-015-0111-3)
Supplement: Additional file 2: Figure S1. — A figure providing a graphical summary of the methods. [file 12920_2015_111_MOESM2_ESM.pdf]

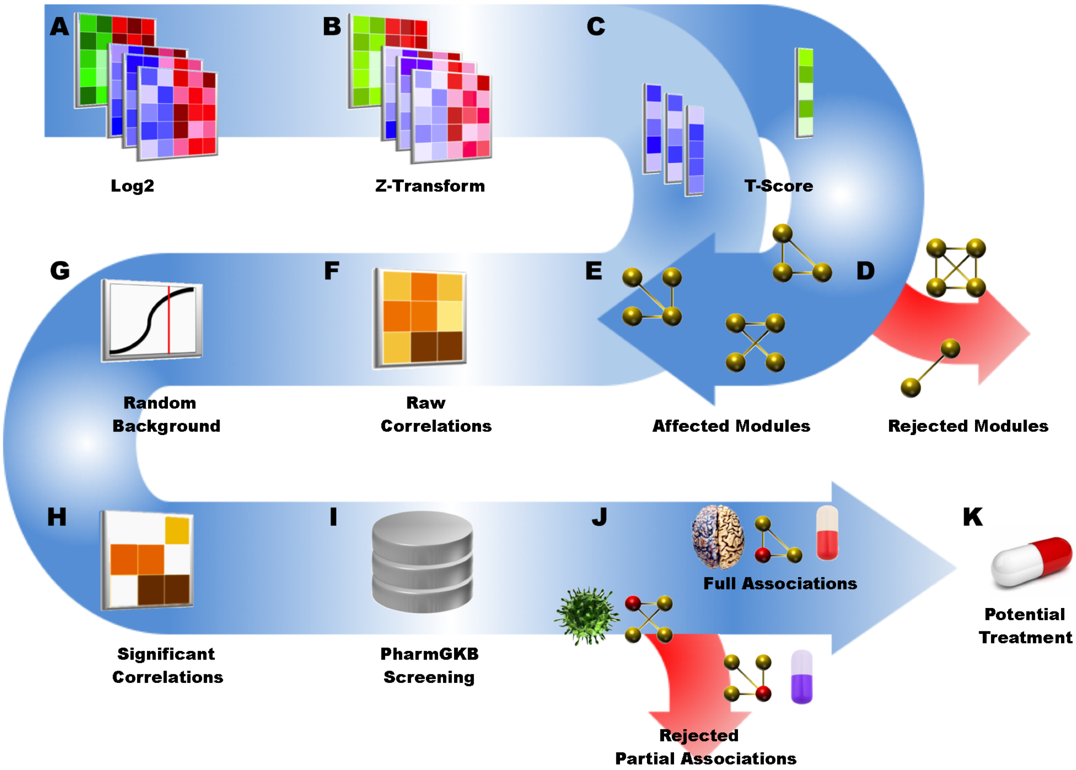

Figure S1 - Summary of Methods

Microarray data for GWI (green), and diseases (blue) with their associated controls (red) are Log2 normalized (A) and then Z-transformed (B). T-Scores are computed across disease and control samples for each gene (C). Average absolute T-Score values are calculated for genes in a given module using GWI data, with modules below threshold being discarded (D) and those above retained for further analysis (E). Partial spearman correlations are calculated between human disease conditions and GWI T-Scores in affected modules (F). Randomized human disease set gene data is compared to intact GWI data in shuffled modules to produce random background correlations for each module (G). Correlations which are not significantly different from random background correlations are removed (H). Remaining genes from modules with significant correlations are run through the PharmGKB database to find associations with diseases and drugs (I). Associations which do not have drug information, or which do not align with diseases shown to correlate with GWI in a specific module are discarded (J). Remaining data reveals potential GWI treatment avenues (K).
